# Conjugation of *Aspergillus flavipes* Taxol with Porphyrin Increases the Anticancer Activity of Taxol and Ameliorates Its Cytotoxic Effects

**DOI:** 10.3390/molecules25020263

**Published:** 2020-01-09

**Authors:** Ashraf S. A. El-Sayed, Maher Fathalla, Marwa A. Yassin, Nabila Zein, Shaima Morsy, Mahmoud Sitohy, Basel Sitohy

**Affiliations:** 1 Enzymology and Fungal Biotechnology Lab, Botany and Microbiology Department, Faculty of Science, Zagazig University, Zagazig 44519, Egypt; m_yassin2007@yahoo.com; 2 Chemistry Department, Faculty of Science, Zagazig University, Zagazig 44519, Egypt; maherfathalla@gmail.com (M.F.); dr.nabila.zein@gmail.com (N.Z.); shaimaa2016@gmail.com (S.M.); 3 Biochemistry Department, Faculty of Agriculture, Zagazig University, Zagazig 44519, Egypt; mzsitohy@hotmail.com; 4 Department of Clinical Microbiology, Infection and Immunology, Umeå University, SE-90185 Umeå, Sweden; basel.sitohy@umu.se; 5 Department of Radiation Sciences, Oncology, Umeå University, SE-90185 Umeå, Sweden

**Keywords:** Taxol, *Aspergillus flavipes*, fluconazole, porphyrin

## Abstract

Taxol is one of the potential anticancer drugs; however, the yield of Taxol and its cytotoxicity are common challenges. Thus, manipulating the Taxol biosynthetic pathway from endophytic fungi, in addition to chemical modification with biocompatible polymers, is the challenge. Four fungal isolates, namely, *Aspergillus flavipes*, *A. terreus*, *A. flavus*, and *A. parasiticus,* were selected from our previous study as potential Taxol producers, and their potency for Taxol production was evaluated in response to fluconazole and silver nitrate. A higher Taxol yield was reported in the cultures of *A. flavipes* (185 µg/L) and *A. terreus* (66 µg/L). With addition of fluconazole, the yield of Taxol was increased 1.8 and 1.2-fold for *A. flavipes* and *A. terreus,* respectively, confirming the inhibition of sterol biosynthesis and redirecting the geranyl phosphate pool to terpenoids synthesis. A significant inhibition of ergosterol biosynthesis by *A. flavipes* with addition of fluconazole was observed, correlating with the increase on Taxol yield. To increase the Taxol solubility and to reduce its cytotoxicity, Taxol was modified via chemical conjugation with porphyrin, and the degree of conjugation was checked from the Thin layer chromatography and UV spectral analysis. The antiproliferative activity of native and modified Taxol conjugates was evaluated; upon porphyrin conjugation, the activity of Taxol towards HepG2 was increased 1.5-fold, while its cytotoxicity to VERO cells was reduced 3-fold.

## 1. Introduction 

Taxol is a diterpenoid alkaloid firstly isolated from *Taxus brevifolia* [1,2] as a strong antimitotic agent, causing cellular arrest at the G2/M phase of tumor cells. The anticancer activity of Taxol has been emphasized towards various tumor cell lines [3], including leukemia, breast, ovarian, and lung cancers [2,4], as well as against polycystic kidney diseases. Taxol production from different sources and their challenges have been addressed extensively on our studies [1,5,6]. Taxol is now being commercially produced by a semi-synthetic method, starting with 10-deacetylbaccatin III [7] extracted from the needles of *T. baccata* and *T. walichiana* growing in Europe and Asia. However, this method is challenged with lower yield, climatic dependence, and seasonal variations (reviewed by [5,8]. Chemical synthesis of Taxol has been emphasized [9,10]; however, the technical complexity and higher expenses are the main challenges stopping this technology from becoming an economically affordable approach [11,12]. 

Deciphering the potency of endophytic fungi for Taxol production opened a new avenue for commercial Taxol production due to their rapid growth, independence on climatic changes, and feasibility of genetic manipulation [5,13]. *Taxomyces andreanae* was the first recognized Taxol-producing endophyte of *Taxus* spp [14], after which an unlimited number of studies became motivated to isolate endophytic fungi with Taxol metabolic-producing potency [5,6,7,8]. Nevertheless, the implementation of these fungi for commercial production of Taxol has been confronted by the loss of Taxol productivity with subculturing and storage [1,6]. Manipulating the Taxol yield by specific inhibitors to modulate the metabolic yield by endophytic fungi was emphasized in our previous study [1]. Similarly, triggering the biosynthesis of secondary metabolites by plants in response to an external effector was also reported [15]. Additionally, using metabolic inhibitors to divert the metabolic flux toward the interested product has also been documented, especially the use of sterol biosynthesis inhibitors to divert the geranyl–geranyl pyrophosphate pool to Taxol biosynthesis, enhancing Taxol yields of up to 50-fold [16].

With the global commercial and clinical values of Taxol, the side effect and required high doses are still the major challenges to this drug, especially in developing countries. Conjugation with various biocompatible polymers is a reliable approach to increase the efficiency of this drug and to reduce its side effects [17]. Porphyrin has been developed as a smart material for drug delivery, which can potentially decrease the side effects of the target drug, increasing its efficient activities. Porphyrins are highly soluble in water and surfactants, and are frequently used in traditional photodynamic therapy. Several types of drugs of nanoscale design have been used together with porphyrins for a variety of applications in imaging and therapy [18,19]. This work was an extension to our previous study [1], to evaluate the Taxol yield by *Aspergillus flavipes* in response to different inhibitors, as well as to increase the bioactivity of extracted Taxol via chemical conjugation with porphyrin rings. In addition, the pharmacokinetic properties of native and modified Taxol were evaluated. 

## 2. Results and Discussion

### 2.1. Taxol Production by the Selected Fungal Isolates in Response to Growth Inhibitors 

**Figure 1:**
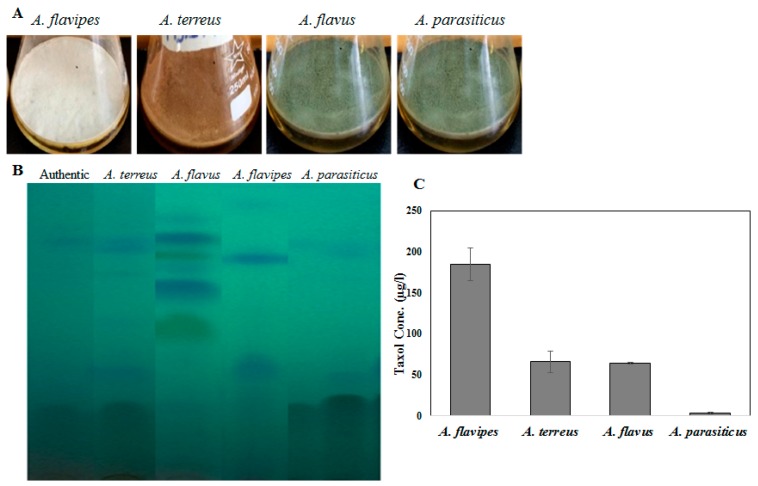
Taxol production by the selected fungal isolates: *Aspergillus flavipes*, *A. terreus*, *A. flavus*, *A. parasiticus*. (**A**) The morphological view of liquid cultures of *Aspergillus flavipes*, *A. terreus*, *A. flavus*, and *A. parasiticus* on PDB medium. (**B**) TLC chromatogram of the experimented fungal isolates. (**C**) Taxol concertation produced by the fungal isolates.

**Figure 2:**
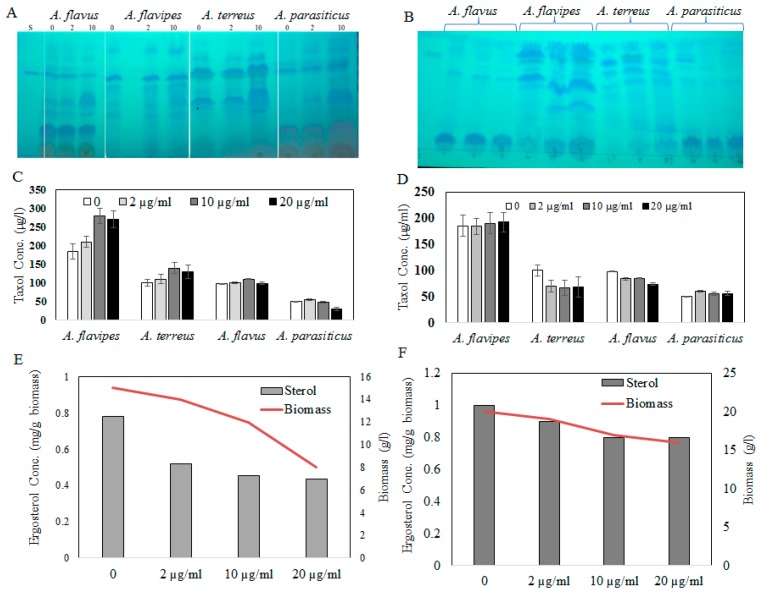
(**A**) TLC chromatogram of Taxol by the fungal isolates in response to fluconazole (0, 2, and 10 µg/mL), and (**B**) AgNO_3_ concentrations (0, 2, and 10 µg/mL). Taxol concentration in response to fluconazole (**C**) and AgNO_3_ (**D**) by the tested fungal isolates by HPLC. Ergosterol concentration of *A. flavipes* (**E**) and *A. terreus* (**F**) in response to fluconazole addition.

Fungal endophytes elevated the hope for mass production of Taxol due to their fast growth, feasibility of fermentation, genetic manipulation, and independence of climatic changes [20]. Nevertheless, the anticipation of fungi for commercial Taxol production has been challenged by their lower yield and drastic loss of Taxol yield with sub-culturing and storage [5,6,8,13,21]. Thus, exploring novel fungal isolates with Taxol-producing potency was the objective. Four fungal isolates, namely, *Aspergillus flavipes* [1], *A. terreus*, *A. flavus*, and *A. parasiticus* [6], were selected based on their higher Taxol productivity. The isolates were grown on potato dextrose broth (PDB) and incubated, and then Taxol was extracted and quantified according to [1,6,8]. The yield of Taxol was emphasized by the TLC and HPLC analyses (Figure 1). The highest Taxol yield was assessed for *A. flavipes* cultures (185 µg/L), followed by *A. terreus* (66 µg/L), *A. flavus* (64 µg/L), and *A. parasiticus* (4 µg/L) (Figure 1). In an endeavor to increase Taxol yield by the selected fungal isolates, different growth inhibitors such as fluconazole and AgNO_3_ were used. After the 5th day of incubation, the fungal cultures were amended with fluconazole and AgNO_3_ (0, 2, 10, and 20 µg/mL final conc.), and then incubation was continued for 20 days before Taxol was extracted and quantified. From the results (Figure 2), fluconazole at 10 µg/mL displayed a strongly positive effect on Taxol yield, estimated as about 1.5-fold compared to control cultures. However, there was no detectable effect on Taxol yield by the tested fungal isolates in response to AgNO_3_ compared to control cultures. The effect of fluconazole as a growth inhibitor on shifting the fungal secondary metabolites’ machinery toward terpenoids biosynthesis has been reported extensively [1,8]. The modulation of Taxol yield by *A. flavipes* and *A. terreus* in response to fluconazole is reasonable, which could be due to inhibition of sterol synthesis as a competitive pathway with Taxol biosynthesis, directing the geranylgeranyl pyrophosphate pool to Taxol biosynthesis. Similar findings reported that AgNO_3_ induced the production of Baccatin III and total taxanes [22], and that at higher fluconazole concentrations, the growth of *A. flavipes* and Taxol yield were significantly reduced, suggesting the inhibition of lanosterol demethylase, causing accumulation of toxic lanosterol intermediates [23].

Sterol and terpenoids are competing pathways for the initial precursor geranyl phosphate; so, the increase of Taxol yield by the experimented fungi with fluconazole incorporation might be explained by blocking the sterol synthesis, accumulating toxic lanosterol intermediates, inhibiting the squalene synthase, thus directing the flux of fungal intermediates towards synthesis of Taxol building blocks “taxadiene”. It has been reported that Taxol production could be induced by growing fungal cells under stress, similarly to other secondary metabolites [24]. To validate the concept of an inhibitory effect of fluconazole on sterol biosynthesis, the ergosterol contents of the fungal biomass were estimated under the same conditions. The cellular ergosterol concentration of *A. flavipes* was slightly reduced upon use of fluconazole; however, there was no detectable effect on ergosterol concentration in response to AgNO_3_, suggesting the inhibitory effect of fluconazole on sterol biosynthesis as a competitive pathway with diterpenoid synthesis, which is consistent with our previous study [5,6,7,8].

### 2.2. Activity of Taxadiene Synthase in Response to Sterol Biosynthesis Inhibitor 

**Figure 3:**
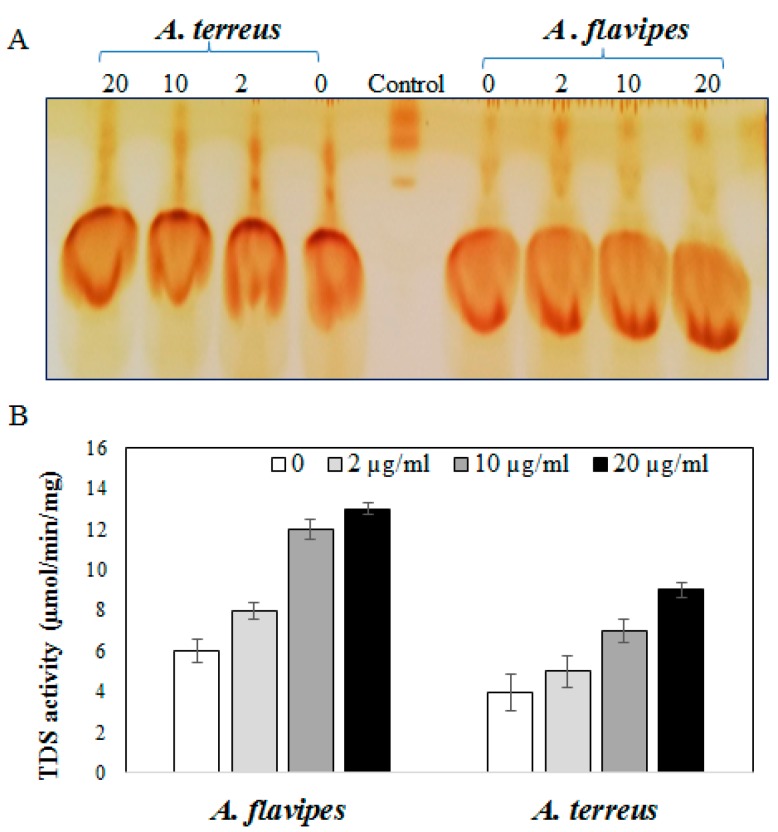
Activity of Taxadiene synthase by *A. flavipes* and *A. terreus* in response to fluconazole (0, 2, 10, and 20 µg/mL). (**A**) TLC chromatogram of residual geranylgeranyl pyrophosphate substrate for TDS from *A. flavipes* and *A. terreus*. (**B**) Activity of Taxadiene synthase from *A. flavipes* and *A. terreus* in response to fluconazole concentrations.

Based on the Taxol yield, *A. flavipes* and *A. terreus* were selected for further validation analysis concerning to activity of Taxadiene synthesis. The fungal cultures treated with fluconazole were homogenized, and enzyme was extracted and assessed based on the residual concentration of geranylgeranyl pyrophosphate [1,8]. The residual concentration of geranylgeranyl pyrophosphate (GGPP) substrate was determined by TLC, and visualized by iodine vapor. From the results, the intensity of residual GGPP spots were reversibly proportional to the activity of the enzyme, which means that the intensity of residual GGPP spots were reduced gradually with the addition of fluconazole compared to control, assuming a significant increase in TDS activity of *A. flavipes* rather than *A. terreus* upon fluconazole addition compared to controls. From the results (Figure 3), the activity of TDS of *A. flavipes* and *A. terreus* was noticeably increased upon addition of fluconazole in a concentration-dependent manner. At a higher concentration of fluconazole (20 µg/mL), the activity of TDS by *A. flavipes* and *A. terreus* was increased about 2.3-fold compared to control cultures (without fluconazole). 

Taken together, based on TDS activity, Taxol yield, and ergosterol concentration, it could be deduced that fluconazole modulates the Taxol biosynthetic machinery by blocking the competitive sterol pathway, which is consistent with our previous results [1,5,6,25]. 

### 2.3. Conjugation of Taxol with Porphyrin

**Figure 4:**
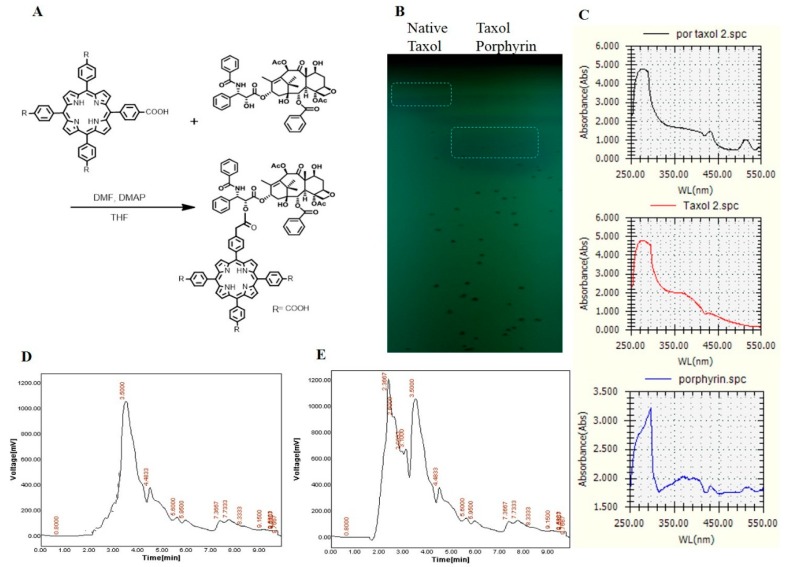
Conjugation of purified Taxol from *A. flavipes* with porphyrin. (**A**) Scheme of chemical conjugation of Taxol with porphyrin, as described in Materials and Methods. (**B**) TLC chromatogram of native Taxol and Taxol–porphyrin conjugates, (**C**) UV spectral analysis of native Taxol, Taxol–porphyrin conjugates, and unbounded porphyrin. The HPLC chromatogram of native Taxol (**D**) and Taxol–porphyrin conjugates (**E**).

The major clinical limitations for Taxol usage are its poor solubility in water and higher dose requirements to get the desired activity. Thus, chemical modification of Taxol and improving the formulation delivery systems are the main targets [26]. Construction of novel drug-therapy systems against cancer has become a research hotspot in biochemistry [27,28], and porphyrin-modified paclitaxel prodrugs are one of the practical technologies for such drug formulations. On the other hand, taking advantage of the fluorescent porphyrin part, the location of nanoparticles in cells could be explicit. Taxol was extracted and purified from *A. flavipes* using a semipreparative-HPLC Puriflash system, as described in Materials and Methods. The purified Taxol was checked and validated chemically by analytical HPLC and TLC, and used for more chemical modification processes. Since the solubility of Taxol and the higher doses required for efficient cancer remedy are the major biotechnological challenges to this drug, improving the structural and biological properties of Taxol was the major objective of this study. The chemical conjugation of Taxol and porphyrin is illustrated in Figure 4. After conjugation, Taxol–porphyrin residues were purified from unbounded Taxol by silica gel column chromatography. The purple-colored Taxol–porphyrin conjugates were collected and dried, and the degree of modification was checked by TLC and UV analysis (Supplementary data). From the results (Figure 4), the Taxol–porphyrin conjugates were eluted at slightly different mobility rates, normalizing to native Taxol. Furthermore, as seen from the UV spectra, a reasonable shift on the absorption maxima of the native Taxol and Taxol–porphyrin conjugates occurred. The lower mobility rate of Taxol–porphyrin conjugates compared to native Taxol suggests the successfulness of the covalent chemical conjugation of Taxol–porphyrin. 

### 2.4. Antiproliferative Activity of Native Taxol and Taxol–Porphyrin Conjugates

**Figure 5:**
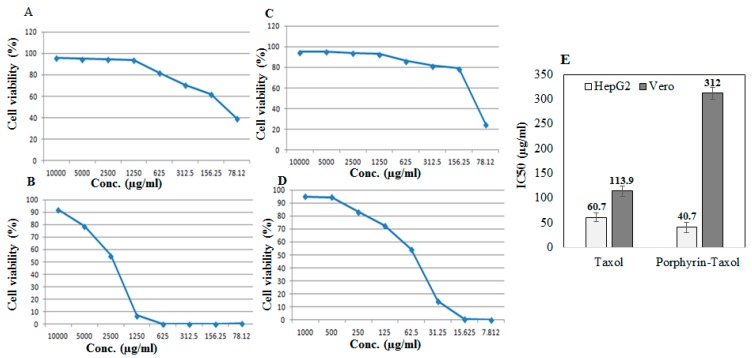
Antiproliferative activity of native Taxol and porphyrin–Taxol conjugates towards HepG2 and VERO normal cell lines. Antiproliferative dose–response curve of native Taxol towards VERO normal cells (**A**), HepG2 tumor cells (**B**), and Porphyrin-Taxol conjugates towards VERO normal cells (**C**) and HepG2 tumor cell lines (**D**). The IC_50_ values of native and Porphyrin-Taxol conjugates towards VERO normal cells and HepG2 tumor cells (**E**).

The antiproliferative activity of the native *A. flavipes* Taxol and Taxol–porphyrin conjugates was evaluated against the liver carcinoma (HEPG2) and African green monkey kidney epithelial cell normal cells (VERO) using MTT assay, as described in Materials and Methods. The antiproliferative activity was expressed from the IC_50_ values (concentration of Taxol required to reduce the growth of 50% of initial number of tumor cells normalizing to control). From the results (Figure 5), the IC_50_ value of the native Taxol and Taxol–porphyrin conjugates was 60.7 µg/mL and 40.7 µg/mL, respectively. So, the antiproliferative activity of Taxol towards HepG2 tumor cells was increased about 1.5-fold upon conjugation with porphyrin. Interestingly, the toxicity of Taxol was reduced about 3-fold to normal VERO cells, as assessed from the IC_50_ values for native Taxol (113.9 µg/mL) and Taxol–porphyrin conjugates (312.2 µg/mL). Thus, it could be deduced that upon conjugation with porphyrin, the antiproliferative activity of Taxol against HepG2 cells was strongly increased; however, its cytotoxic activity was significantly reduced. This increase in anticancer activity with profound reduction to its cytotoxic effect could be ascribed to the molecular improvement on solubility of Taxol by increasing its surface hydrophilicity [29].

### 2.5. Biochemical Profile of Mice in Response to Native and Taxol–Porphyrin Conjugates

The biochemical profile of mice in response to a single dose of native and Taxol–porphyrin conjugates was evaluated. From the data (Table 1), all of the measured biochemical parameters were slightly affected in response to Taxol–porphyrin conjugates, unlike to the significant changes due to unmodified Taxol, suggesting the mitigating and ameliorating effect of porphyrin on the Taxol compounds. A significant decrease in kidney enzymes, especially alanine amino transferase (ALT) and aspartate amino transferase (AST), in contrast to the significant decrease in alkaline phosphatase activity in response to unmodified Taxol was observed.

## 3. Materials and Methods

### 3.1. Fungal Isolates, Media Preparation, and Screening for Taxol Production 

Four different fungal isolates, namely, *Aspergillus flavipes, A. terreus, A. flavus,* and *A. parasiticus,* were selected in the current study based on their yield of Taxol from our previous studies [6,8]. The current fungal isolates were morphologically identified based on their macro and microscopical features [30]. *Aspergillus flavipes* was isolated from Egyptian soil samples [1,31,32,33,34,35,36], while *A. terreus, A. flavus*, and *A. parasiticus* were isolated as endophytes from *Podocarpus gracilior* [6,8]. The isolates were maintained on potato dextrose agar (PDA) medium [37] (extract of 250 g potato, 20 g glucose, and 20 g agar, dissolved in 1 L tap water). The fungal isolates were screened for Taxol production by inoculating 2 agar plugs of 5 mm from 6-day-old pure cultures of each fungal isolate into 50 mL/250 mL Erlenmeyer flask of potato dextrose broth (PDB) [38]. Three biological replicates from each fungal isolate were prepared. Flasks of PDB media without fungal inocula were used as negative controls at the same conditions. After incubation, mycelia were filtered from the liquid medium using sterile cheesecloth to remove the fungal mycelia, and fatty acids were precipitated from the filtrate by 0.03% sodium bicarbonate. Taxol was extracted with double volume of dichloromethane (DCM) and separated by TLC analysis as described previously [1,2,3,4,5,6]. 

### 3.2. Taxol Purification by Preparative-HPLC System

The extracted Taxol was purified by a semipreparative technique with Puriflash 4100 system (Interchim, Montluҫon, France) with HPLC quaternary pump, a PDA–UV-Vis detector 190–840 nm, a fraction collector, and a sample loading module. For system controlling and process monitoring, Interchim software 5.0 (Montluçon, France) was used. The crude extract (3 g) was dissolved in 50 mL methanol, and then dissolved on a small amount of stationary phase (silica). The deposit solvent was evaporated with a rotary evaporator until dry powder was obtained. The fractionation was performed using automated flash chromatography (Puriflash 4100, Interchim), with pre-packed INTERCHIM PF-30SI-HP (Montluçon, France) (30 µm silica gel) columns at a flow rate of 15 mL/min, a pressure 22 bar, using a gradient of methanol and DCM for the elution.

### 3.3. TLC, UV, and HPLC Analyses

The sample was fractionated and identified by TLC [6,8,39] using Merck 1 mm (20 × 20 cm) pre-coated silica gel plates. The solvent systems chloroform/methanol (7:1, *v*/*v*), chloroform/ acetonitrile (7:3 *v*/*v*), ethylacetate/2-propanol (95:5 *v*/*v*), methylene chloride/tetrahydrofuran (6:2 *v*/*v*), and methylene chloride/methanol/dimethyl-formamide (90:9:1 *v*/*v*/*v*) were used. Taxol spots were detected by UV illumination (Min-UVIS, DESAGA, Heidelberg, Germany) at 254 nm regarding the R_f_ value of authentic Taxol (Cat. # T7402) giving blue-colored spots. The intensities of the target spots were assessed by the ImageJ software package (https://imagej.nih.gov/ij/download.html). The putative Taxol spots of silica were scraped off from the plate and eluted with DCM. The purity and concentration of eluted Taxol samples were analyzed by HPLC (Agilent Technol, G1315D, USA) with an RP- C18 column (Eclipse Plus C18 4.6 × 150 mm, 3.5 µm, # 959963-902). The isocratic mobile phase was acetonitrile/water (52:48 *v*/*v*/*v*) at a flow rate of 1.0 mL/min. The sample and mobile phase were filtered, injected into the column, and analyzed for 20 min. The fractions were scanned from 200 to 500 nm by a photodiode array detector (DAD). The identity and concentration of the Taxol sample were confirmed from the retention time and absorption peak area at 228 nm [21].

The UV spectra of recovered Taxol were scanned at λ 200–300 nm (Spectrophotometer, RIGOL, ultra-3000 Series, Hyperions ApS Sandholmvej, DK-3450 Allerød, Denmark) with blank medium as negative control. The samples were dissolved in methanol. 

### 3.4. LC–MS Analyses

The spots of silica gel containing Taxol on the TLC sheets were scraped-off and eluted with methanol [6]. The chemical structure of extracted fungal Taxol was validated from LC–MS/MS analyses (Waters ACQUITY, LC-MS, XEVO-TQD#QCA423, Waters, USA). The HPLC portion was run isocratically with acetonitrile/water (49:51) as the mobile phase. The samples in 100% methanol were infused into the mass spectrometer through a reverse-phase C18 column at a flow rate of 0.2 mL/min with a column temperature of 27.8 °C and a spray voltage of 3 kV by the loop injection method. The MS scanning ranged from 100 to 1000 *m*/*z.*

### 3.5. Eliciting the Taxol Yield of Selected Fungal Isolate with Growth Inhibitors

The effect of fluconazole on inducing the Taxol yield by the selected fungal isolate was evaluated [1]. The Taxol production media PDB was supplemented with different concentrations of fluconazole (0, 2, 10, and 20 µg/mL) after 5 days of preincubation. The cultures were re-incubated for 15 days, and then the filtered using sterile cheesecloth to remove the fungal mycelia. Taxol was extracted and assessed by TLC and HPLC, and the activity of Taxadiene synthase “rate-limiting enzyme” of Taxol biosynthesis was determined [8].

### 3.6. Taxadiene Synthase (TDS) Activity Assay 

After incubation at the desired conditions, the fungal cultures were filtered and the collected mycelia were washed using sterile distilled water. Five grams of the fungal fresh biomass were pulverized in liquid nitrogen, dispensed in 10 mL of 30 mM, pH 8.0 HEPES buffer with 5 mM sodium ascorbate, 5 mM DTT, 5 mM Na_2_S_2_O_5_, 15 mM MgCl_2_, 10% (*v*/*v*) glycerol, and 1% (*w*/*v*) polyvinylpolypyrrolidone. The homogenate was centrifuged at 8000 rpm for 15 min; then, the supernatant was used as a source of crude enzyme. The activity of Taxadiene synthase (TDS) was assessed [40] with slight modifications [8]. The reaction mixture contained 500 µL of the crude enzyme, 1 mL of 30 mM HEPES buffer (pH 8.0), and 50 µL of 1 mM geranylgeranyl pyrophosphate (GGPP). The reaction was incubated at 32 °C for 1 h, and terminated by 50 µL of EDTA (0.5 M, pH 8.0). The GGPP concentration was determined by TLC using silica gel plates 60 F_254_ (Merck KGaA, Darmstadt, Germany) with developing solvent system propan-2-ol, ammonia, and water (9:3:1) regarding to the authentic GGPP (Cat#. G6025) after visualization by vapor iodine [41]. The intensity of GGPP spots was determined by ImageJ software [1,6,8].

### 3.7. Total Sterol Assay 

Total cellular sterols were extracted according to Breivik and Owades [42]. Briefly, 1.5 g homogenized fungal tissues was homogenized in alcoholic potassium hydroxide solution (4 mL), in 100 mm sterile borosilicate glass screw-cap tubes, with incubation at 85 °C for 1 h, and then the tubes were cooled to room temperature. Sterols were then extracted with n-heptane with vigorous mixing for 3 min. The heptane layer was transferred into a clean borosilicate glass screw-cap tube and stored at −20 °C until use. Prior to analysis, the sterol extract was diluted 5-fold in 100% ethanol and scanned at 240–300 nm (RIGOL, ultra-3000 Series, Hyperions ApS Sandholmvej, DK-3450 Allerød, Denmark). The presence of ergosterol and sterol intermediate 24(28) dehydro-ergosterol in the extract was confirmed from the distinctive four-peaked curve. A flat line indicated the absence of detectable ergosterol, and the amount of ergosterol was calculated as described previously [1,8].

### 3.8. Conjugation of Taxol with Porphyrin 

The chemical conjugation of Taxol with porphyrin was conducted according to [17,29,43], as illustrated in Figure 4A. Briefly, tetracarboxyphenyl porphyrin (0.3 mM), *N*,*N*′-dicyclohexylcarbodi imide (DCC) (0.25 mM), 4-dimethylaminopyridine (DMAP) (0.1 mM), and Taxol (0.2 mM) were dissolved in tetrahydrofuran (THF) (10 mL), and the mixture was then stirred in the dark at room temperature under nitrogen conditions for 72 h. The solution was filtered, and the filtrate was dried under the reduced pressure to remove the solvent. The residues were dissolved in chloroform (50 mL) and washed with water (3 × 50 mL), and the organic phase was then dried over Na_2_SO_4_. The solvent was removed under the reduced pressure, and the crude product was purified by column chromatography to give Taxol–porphyrin conjugates as a purple solid pellet.

### 3.9. Antiproliferative Activity of Native Taxol and Taxol–Porphyrin Conjugates

The antiproliferative activity of native fungal Taxol and Taxol–porphyrin conjugates against liver carcinoma (HEPG2) and normal African green monkey kidney cell (VERO) was evaluated (VACSERA, Cairo, Egypt). The tumor cell viability was assessed with 3-(4,5-dimethylthiazol-2-yl)- 2,5-diphenyl tetrazolium bromide (MTT) assay [44]. The 96-well plate was seeded with 10^3^ cells/well, incubated for 24 h, supplemented with different concentrations of native and Taxol–porphyrin conjugates, and re-incubated for 48 h. Twenty microliters (μL) of MTT dye was added, incubated for 6 h, and the developed purple color formazan complex was measured at λ570 nm. The IC_50_ value was expressed by the concentration of Taxol reducing the growth of 50% of initial number of cells, normalizing to positive control (Paclitaxel, No. 23958/2005, Cipla, India).

### 3.10. Biochemical Profile of Mice in Response to Native and Taxol–Porphyrin Conjugates

The biochemical toxicity of the native and Taxol–porphyrin conjugates was assessed in vivo using male mice (25 g, 30 days old). The experiments were conducted according to the guidelines of the Institutional Animal Care and Use Committee (IACUC) at Faculty of Medicine, Zagazig University, following the National Institute of health guidelines under protocol 15-08-263. After acclimatization for 3 days, a single dose (100 μL) of the native and Taxol–porphyrin conjugates (0.4 µg/mL) was injected into the tail vein of mice and acclimated for 20 days. Each treatment group had five mice. Normal mice and primate plasma of injected mice were used as negative and positive controls, respectively. Blood samples were collected intervally to assess the biochemical and hematological parameters. The cytotoxicity of Taxol and Taxol–porphyrin conjugates was determined based on biochemical parameters such as ALT, AST, ALP, total protein, albumin, globulin, urea, and creatinine (Spectrum Co., Cairo, Egypt).

### 3.11. Deposition of the Fungal Isolates

The potent Taxol-producing fungal isolates *Aspergillus flavipes*, *A. terreus*, and *A. flavus* were molecularly identified based on their sequences of 18S-ITS-28S regions and deposited in GenBank with accession numbers JF831014.1, MH156195, and MF377553, respectively. 

### 3.12. Statistical Analyses

All experiments were conducted in triplicates and the data were analyzed using one-way ANOVA (https://goodcalculators.com/one-way-anova-calculator/) and represented by the means and standard deviations.

## 4. Conclusions

In conclusion, Taxol productivity by the four selected fungal isolates, namely, *A. flavipes*, *A. terreus*, *A. flavus*, and *A. parasiticus*, was evaluated in response to fungal growth inhibitors, fluconazole and silver nitrate. Maximum Taxol yield was detected with *A. flavipes* cultures, and its yield was duplicated upon addition of fluconazole. To increase the solubility of Taxol, it was conjugated with porphyrin, and the conjugation efficiency was evaluated by TLC and UV spectral analysis. The anticancer activity of Taxol was increased about 2-fold, upon conjugation with porphyrin, towards HepG2. Additionally, the cytotoxicity of Taxol–porphyrin conjugates on normal VERO cells was strongly reduced compared to native Taxol.

## Figures and Tables

**Table 1:** Biochemical analysis of mice in response to Taxol and porphyrin–Taxol conjugates (por–Taxol).

Test		12 h		24 h		48 h	
	Control	Por–Taxol	Taxol	Por–Taxol	Taxol	Por–Taxol	Taxol
Alanine amino transferase (ALT) (U/L)	221.5	252	248	220	231	185	150
Aspartate aminotransferase (AST) (U/L)	310	334	178	616	323	216	125
Alkaline phosphatase (ALP) (U/L)	276	871	714	1067	682	214	702
Total protein (g/dl)	8.2	5.67	5.46	9.2	9.5	7.9	5.14
Albumin (g/dl)	5	4.73	3.9	5.72	6.2	5.58	3.01
Globulin (g/dl)	3.2	2.94	1.56	3.48	3.3	1.32	2.13
Albumin/Globulin ratio	1.6	0.93	2.5	1.64	1.88	1.95	1.41
Urea (mg/dl)	12.8	11.3	26	11.1	28.4	16.2	20.9
Creatinine (mg/dl)	0.8	0.84	0.46	0.99	2.09	0.6	0.85

## Supplementary Materials

The supplementary materials are available online.

## References

[1] El-Sayed A.S.A., Ali D.M.I., Yassin M.A., Zayed R.A., Ali G.S. (2019). Sterol inhibitor “Fluconazole” enhance the Taxol yield and molecular expression of its encoding genes cluster from Aspergillus flavipes. Process Biochem..

[2] Wani M.C., Taylor H.L., Wall M.E., Coggon P., McPhail A.T. (1971). Plant antitumor agents. VI. The isolation and strcture of taxol, a novel antileukemic and antitumor agent from Taxus brevifolia. J. Am. Chem. Soc..

[3] Suffness M., Cordell G.A. (1985). Antitumor alkaloids. Alkaloids.

[4] Rowinski E.K., Cazenave L.A., Donehower F.C. (1990). Taxol: A novel investigational antimicrotubule agent. J. Natl. Cancer Inst..

[5] El-Sayed A.S.A., Abdel-Ghany S.E., Ali G.S. (2017). Genome editing approaches: Manipulating of lovastatin and taxol synthesis of filamentous fungi by CRISPR/Cas9 system. Appl. Microbiol. Biotechnol..

[6] El-Sayed A.S.A., Safan S., Mohamed N.Z., Shaban L., Ali G.S., Sitohy M.Z. (2018). Induction of Taxol biosynthesis by Aspergillus terreus, endophyte of Podocarpus gracilior Pilger upon intimate interaction with the plant endogenous microbes. Process Biochem..

[7] Barboni L., Gariboldi P., Torregiani E., Appendino G., Gabetta B., Zini G., Bombardelli E. (1993). Taxanes from the needles of Taxus wallichiana. Phytochemistry.

[8] El-Sayed A.S.A., Mohamed N.M., Safan S., Yassin M.A., Shaban L., Shindia A., Ali G.S., Sitohy M. (2019). Restoring the Biosynthetic Machinery of Taxol of Aspergillus terreus via cocultivation with the endophytic microbiome of Podocarpus gracilior Pilger. Sci. Rep..

[9] Holton R.A., Somoza C., Kim H.B. (1994). First total synthesis of taxol.1. Functionalization of the B ring. J. Am. Chem. Soc..

[10] Nicolaou K.C., Yang Z., Liu J.J., Ueno H., Nantermet P.G., Guy R.K., Claiborne C.F., Renaud J., Couladouros E.A., Paulvannan K. (1994). Total synthesis of taxol. Nature.

[11] Borman S. (1994). Scientists mobilize to increase supply of the anticancer drug taxol. Chem. Eng. News.

[12] Borman S. (1994). Total synthesis of anticancer agent taxol achieved by two different routes. Chem. Eng. News.

[13] Stierle A., Strobel G., Stierle D., Grothaus P., Bignami G. (1995). The search for a taxol producing microorganism among the endophytic Fungi of the Pacific Yew, Taxus brevifolia. J. Nat. Prod..

[14] Stierle A., Strobel G., Stierle D. (1993). Taxol and taxane production by Taxomyces andreanae, an endophytic fungus of Pacific yew. Science.

[15] Baldi A., Farkya S., Jain A., Gupta N., Mehra R., Datta V. (2009). Enhanced production of podophyllotoxins by co-culture of transformed Linum album cells with plant growth-promoting fungi. Pure Appl. Chem..

[16] Li J.Y., Sidhu R.S., Ford E., Hess W.M., Strobel G.A. (1998). The induction of taxol production in the endophytic fungus- Periconia sp. from Torreya grandifolia. J. Ind. Microbiol..

[17] Zheng X., Li Z., Chen L., Xie Z., Jing X. (2016). Self-Assembly of Porphyrin–Paclitaxel Conjugates Into Nanomedicines: Enhanced Cytotoxicity due to Endosomal Escape. Chem. Asian J..

[18] Dougherty T.J., Gomer C.J., Henderson B.W., Jori G., Kessel D., Korbelik M., Johan M., Qian P. (1998). Photodynamic therapy. J. Natl. Cancer Inst..

[19] Huynh E., Lovell J.F., Helfield B.L., Jeon M., Kim C., Goertz D.E., Wilson B.C., Zheng G. (2012). Porphyrin shell microbubbles with intrinsic ultrasound and photoacoustic properties. J. Am. Chem. Soc..

[20] Exposito O., Bonfill M., Moyano E., Onrubia M., Mirjalili M.H., Cusido R.M., Palazon J. (2009). Biotechnological production of taxol and related taxoids: Current state and prospects. Anticancer Agents Med. Chem..

[21] Nims E., Dubois C.P., Roberts S.C., Walker E.L. (2006). Expression profiling of genes involved in paclitaxel biosynthesis for targeted metabolic engineering. Metab. Eng..

[22] Bringi V., Kakrade P.G., Prince C.L., Roach B. (1997). Enhanced Production of Taxanes by Cell Cultures of Taxus Species. Patent.

[23] Chong H.S., Campbell L., Padula M.P., Hill C., Harry E., Li S.S., Wilkins M.R., Herbert B., Carter D. (2012). Time-course proteome analysis reveals the dynamic response of Cryptococcus gattii cells to Fluconazole. PloS ONE.

[24] Fett-Neto A.G., Melanson S.J., Nicholson S.A., Pennington J.J., DiCosmo F. (1994). Improved taxol yield by aromatic carboxylic acid and amino acid feeding to cell cultures of Taxus cuspidata. Biotechnol. Bioeng..

[25] Siegel M.R. (1981). Sterol-Inhibiting Fungicides: Effects on Sterol Biosynthesis and sites of action. Plant Dis..

[26] Nanda R., Sasmal A., Nayak P.L. (2011). Preparation and characterization of chitosan–polylactide composites blended with Cloisite 30B for control release of the anticancer drug paclitaxel. Carbohydr. Polym..

[27] Brigger I., Dubernet C., Couvreur P. (2013). Nanoparticles in cancer therapy and diagnosis. Adv. Drug Deliv. Rev..

[28] Li Y., Shi J. (2014). Hollow-structured mesoporous materials: Chemical synthesis, functionalization and applications. Adv. Mater..

[29] Yang Y., Zhang Y.M., Chen Y., Chen J.-T., Liu Y. (2016). Polysaccharide-based Noncovalent Assembly for Targeted Delivery of Taxol. Sci. Rep..

[30] Raper K.B., Fennell D.I. (1965). The Genus Aspergillus.

[31] El-Sayed A.S.A., Khalaf S.A., Abdel Hamid G., El-Batrik M.I. (2015). Screening, morphological and molecular identification of cystathionine γ-lyase producing fungi. Acta Biol. Hung..

[32] El-Sayed A.S.A., Iqrar I., Ali R., Norman D., Brennan M., Ali G.S. (2018). A glucanolytic Pseudomonas sp. associated with Smilax bona-nox L. displays strong activity against Phytophthora parasitica. Microbiol. Res..

[33] El-Sayed A.S.A., Ruff L.E., Ghany S.E.A., Ali S.E.A., Esener S. (2017). Molecular and spectroscopic characterization of Aspergillus flavipes and Pseudomonas putida L-Methionine γ-Lyase in vitro. Appl. Biochem. Biotechnol..

[34] El-Sayed A.S.A., Shindia A.A., AbouZaid A.A., Yassin A.M., Ali G.S., Sitohy M. (2019). Biochemical characterization of peptidylarginine deiminase-like orthologs from thermotolerant Emericella dentata and Aspergillus nidulans. Enzym. Microb. Technol..

[35] El-Sayed A.S.A., George N.M., Bolbol A.A., Mohamed M.S. (2019). Purification and biochemical characterization of Aspergillus terreus ornithine decarboxylase: Curcumin is a potent enzyme inhibitor. Molecules.

[36] El-Sayed A.S.A., Ali G.S. (2020). Aspergillus flavipes is a novel efficient biocontrol agent of Phytophthora parasiticus. Biol. Control.

[37] Bilgrami K.S., Verma R.N. (1981). Physiology of Fungi.

[38] Heinig U., Scholz S., Jennewein S. (2013). Getting to the bottom of Taxol biosynthesis by fungi. Fungal Divers..

[39] Li J.Y., Strobel G., Sidhu R., Hess W.M., Ford E.J. (1996). Endophytic taxol-producing fungi from bald cypress, Taxodium distichum. Microbiology.

[40] Hezari M., Lewis N.G., Croteau R. (1995). Purification and characterization of taxa-4(5), 11(12)-diene synthase from Pacific yew (Taxus brevifolia) that catalyzes the first committed step of taxol biosynthesis. Arch. Biochem. Biophys..

[41] Artz J., Wernimont A.K., Dunford J.E., Schapira M., Dong A., Zhao Y., Lew J., Russell R.G.G., Ebetino F.H., Oppermann U. (2011). Molecular characterization of a novel Geranylgeranyl Pyrophosphate Synthase from Plasmodium parasites. J. Biol. Chem..

[42] Breivik O.N., Owades J.L. (1957). Spectrophotometric semi-microdetermination of ergosterol in yeast. Agric. Food Chem..

[43] Fathalla M., Strutt N.L., Sampath S., Katsiev K., Hartlieb K.J., Bakr O.M., Stoddart J.F. (2015). Porphyrinic supramolecular daisy chains incorporating pillar[5]arene-viologen host-guest interactions. Chem. Commun..

[44] Cory A.H., Owen T.C., Barltrop J.A. (1991). Use of an aqueous soluble tetrazolium/formazan assay for cell growth assays in culture. Cancer Commun..

