# Supplementary material for: Conjugation of Aspergillus flavipes Taxol with Porphyrin Increases the Anticancer Activity of Taxol and Ameliorates Its Cytotoxic Effects
Source: Molecules. 2020 Jan 9;25(2):263. doi: 10.3390/molecules25020263 (PMC7024149; doi:10.3390/molecules25020263)
Supplement: Supplementary file 1 [file molecules-25-00263-s001.pdf]

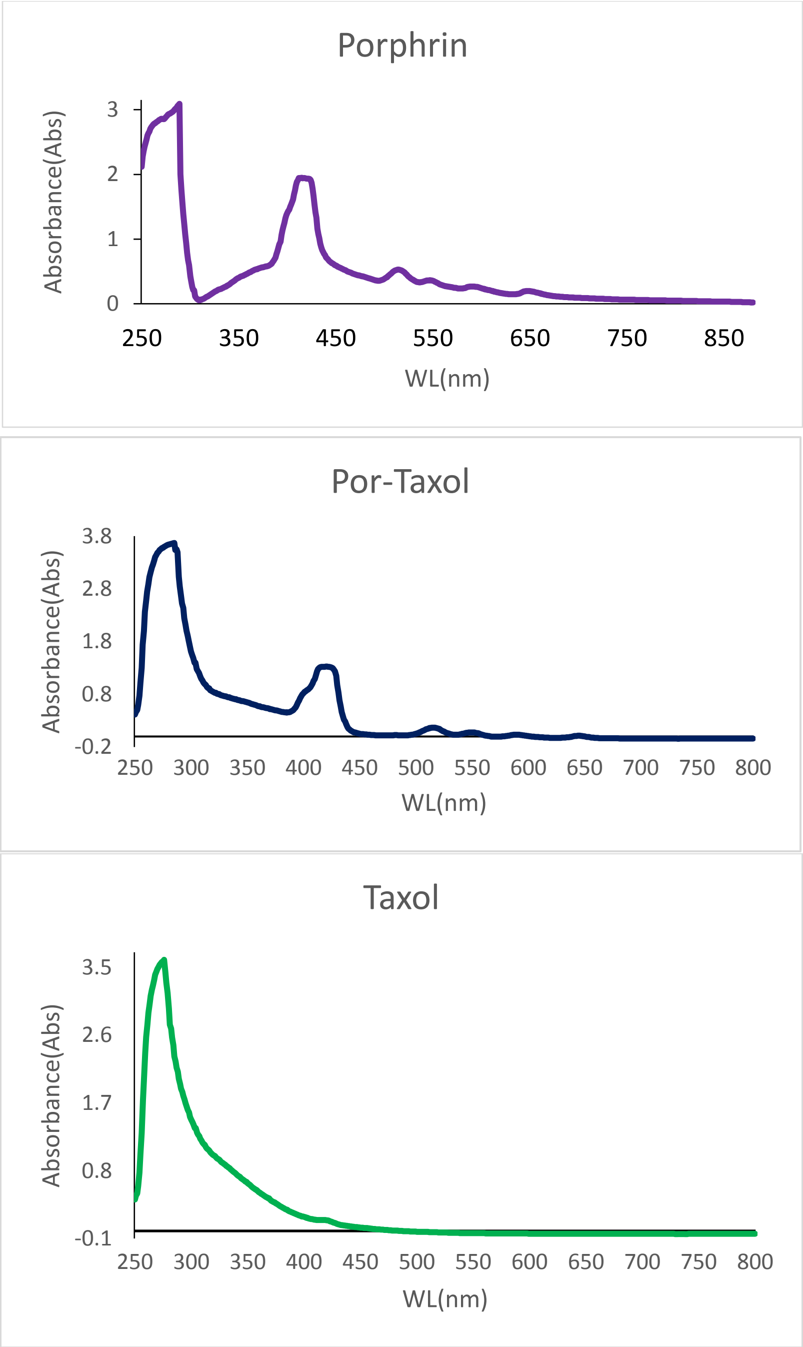

Figure S1. UV-VIS spectra of tetracarboxyphenylporphyrin (top), taxol-porphyrin conjugate (middle) and taxol (bottom) in THF.
